# The Impact of Endograft Selection on Outcomes Following Treatment Outside of Instructions for Use (IFU) in Endovascular Abdominal Aortic Aneurysm Repair (EVAR)

**DOI:** 10.7759/cureus.14841

**Published:** 2021-05-04

**Authors:** Ian P Barry, Luke P Turley, Daniela L Mwipatayi, Angel Thomas, Mariah T Mwipatayi, Bibombe P Mwipatayi

**Affiliations:** 1 Vascular and Endovascular Surgery, Royal Perth Hospital, Perth, AUS; 2 Surgery, Royal College of Surgeons in Ireland, Dublin, IRL; 3 Medicine, University of Medicine and Health Sciences, Royal College of Surgeons in Ireland, Dublin, IRL; 4 Curtin Medical School, Faculty of Health Sciences, Curtin University, Perth, AUS; 5 Medicine, University of Buckingham Medical School, Buckingham, GBR; 6 Vascular and Endovascular Surgery, University of Western Australia, Perth, AUS

**Keywords:** abdominal aortic aneurysm, endovascular repair, instructions for use, endoleak, mortality

## Abstract

Background

Endovascular aneurysm repair (EVAR) has become the treatment modality of choice in patients with abdominal aortic aneurysms. This has resulted in endograft utilization within instructions for use (IFU) and in cases with proximal neck anatomy outside of IFU.

Purpose

To identify whether graft selection influences outcomes following EVAR outside of IFU.

Methodology

A retrospective analysis was conducted from previously published data for 636 patients, collated from the Endurant Stent Graft Natural Selection Global Post-Market Registry (ENGAGE) and the Global Registry for Endovascular Aortic Treatment (GREAT). Patients were recruited into the ENGAGE registry between 2009 and 2011 and into the GREAT registry between August 2010 and October 2016. In ENGAGE, they received the Medtronic Endurant stent graft (Medtronic Vascular Inc, Dublin, Ireland) for infrarenal AAA repair while patients analyzed in GREAT received the Gore Excluder stent-graft (W. L. Gore & Associates, Flagstaff, Arizona). Analyses were performed to evaluate all-cause mortality, aneurysm-related mortality, endoleak occurrence, and surgical reintervention rates between the two cohorts.

Results

Of the 636 patients, 225 were from ENGAGE (mean age 73 years) and 411 were from GREAT (mean age 75 years). 17.8% were treated outside of IFU in the ENGAGE registry, while 12.4% were treated outside IFU in the GREAT cohort. Five-year freedom from all-cause mortality was similar in both cohorts (65.6% vs. 63.8%). The rate of type IA endoleak development was lower in the Excluder cohort, although this may have been impacted by the fact that only endoleaks that underwent reintervention were recorded within GREAT analysis (Endurant 10.6% vs. Excluder 7.0%). The reintervention rate was 16% at five years following the Endurant aortic graft while it was 13.3% at five years with the Excluder.

Conclusion

Treatment outside of IFU, be it with a suprarenal or an infrarenal fixation device, is associated with worse outcomes. This analysis reinforces the importance of the consideration of either fenestrated or open repair in those aneurysms that fail to satisfy IFU while endovascular repair in such a setting should be reserved as a last resort strategy.

## Introduction

Endovascular aneurysm repair (EVAR) has become the predominant treatment modality for abdominal aortic aneurysms (AAAs) since its introduction over 20 years ago [1]. The change in management was promoted by several short-term benefits, including reduced hospital stay, a swift return to independent living, and low early overall mortality as compared to open aortic repair (OAR) [2-4]. However, when comparing EVAR to OAR, the mid- to long-term outcomes did not convey a similar advantage [5]. Factors such as stent migration or endoleaks appeared to be the most culpable agents, with the development of subsequent aneurysm repressurization and rupture [6-7].

In response to these issues, graft manufacturers sought to advance the technology and examine risk factors for the development of endoleaks in an attempt to prevent future complications. This led to further development of the "instructions for use" (IFU) criteria. These are a specific set of criteria, provided by the manufacturers, which describe particular aneurysm morphology that should be satisfied to ensure the safe and appropriate utilization of endoprostheses in the correct environment. Criteria vary between grafts, with significant differences noted in those stents involving suprarenal fixation as compared to infrarenal fixation. The IFU generally incorporate factors such as a minimum aortic neck diameter, a minimum aortic neck length, and a maximum aortic neck angulation.

Two of the most widely utilized endoprostheses are the Endurant (Medtronic Vascular Inc, Dublin, Ireland), and the Excluder (W. L. Gore & Associates, Flagstaff, Arizona). Each device has different accepted thresholds for these morphologic features as expressed in their instructions for use (IFU) [8]. The variation in thresholds for device application is likely impacted by the site of proximal stent fixation; Endurant is a suprarenal device as compared to Excluder, an infrarenal device.

The ongoing technological advancements in endograft design and delivery have resulted in a propensity to utilize these stents outside of IFU. The aim of this study is to assess whether graft selection, comparing a suprarenal device and an infrarenal device, influences outcomes following endoluminal AAA repair outside of the IFU.

## Materials and methods

Data source

Previously published data were obtained and subsequently collated from two sources; the Endurant Stent Graft Natural Selection Global post-market registry (ENGAGE) and the Global Registry for Endovascular Aortic Treatment (GREAT) [9-10].

ENGAGE (Clinicaltrials.gov identifier, NCT00870051) was designed to augment the knowledge base about endovascular aortic aneurysm repair in a real-world population implanted with the Medtronic stent graft system (Endurant) [9]. Patient enrollment in the ENGAGE registry occurred between 2009 and 2011, with a follow-up duration of 10 years. A total of 1263 patients from 79 international centers were enrolled with minimal inclusion criteria. Selected exclusion criteria included the probability of non-adherence to follow-up requirements and concurrent participation in another trial that might confound results [9]. Data on outcomes following five years of follow-up were utilized within this analysis.

GREAT (Clinicaltrials.gov identifier, NCT01658787) was designed for the collection of data on the management of serious adverse events and follow-up patterns after the implantation of all Gore® aortic endografts (W.L. Gore Associates) [10]. Patient enrolment in the GREAT registry occurred between August 2010 and October 2016. Over 5,000 patients from 114 international centers were enrolled, with broad inclusion criteria and minimal exclusion criteria, reflecting the real-world use of devices, with a follow-up duration of 10 years [10]. Data on outcomes following five years of follow-up were utilized within this analysis.

Endoluminal prosthesis

The Endurant endograft, available since 2008, has a two-piece design with a nitinol M-shaped stent skeleton, which is covered with polyester fabric. Proximal fixation is augmented by a suprarenal stent with anchoring pins. The Endurant II was introduced in December 2011 and differed from its predecessor regarding additional radiopaque markers and reduction in the delivery system profile. A later design (the Endurant IIs) included a three-piece design, although this was only made available in 2014. It is not included in this analysis, as inclusion in the ENGAGE registry ended in 2011 [8].

The Excluder endoprosthesis is a modular bifurcated system that has been available since 1997. The main body features eight nitinol anchors for infrarenal fixation while the endografts’ nitinol stent frame is covered by polytetrafluoroethylene (PTFE). In 2004, a structural change to the device was made with the addition of a low permeability expanded PTFE sleeve to the graft composition, owing to high rates of sac growth with the original device. In 2010, the C3 Delivery System appeared, but no graft modifications were made [8].

The Endurant is suitable for infra-renal necks ranging from 19-32 mm, allowing the treatment of infrarenal necks ≥ 10 mm in length if the infrarenal angulation is ≤ 60^o^ and the suprarenal angulation is ≤ 45^o^. It is also suitable for a neck length of ≥ 15 mm with an infrarenal angulation of ≤ 75^o^ and a suprarenal angulation of ≤ 60^o^. The Excluder is more conservative and is suitable for infra-renal necks ranging from 19-29 mm in diameter and ≥ 15 mm in length, with an infrarenal angulation of ≤ 60^o^ [8].

Inclusion criteria

All patients who underwent EVAR for AAA outside of the IFU in the two aforementioned databases were included for analysis. In the ENGAGE registry, IFU recommended an adequate iliac or femoral access vessel morphology compatible with vascular access of the graft to the aorta; proximal aortic neck length of >10 mm with insignificant calcification and thrombus and <60^o^ of infrarenal and <45^o^ of suprarenal angulation or a proximal neck length of 15 mm or greater with insignificant calcification and thrombus with <75o of infrarenal and <60o of suprarenal angulation; distal fixation length of >15 mm or more; aortic neck diameters with a range of 19 to 32 mm; and iliac diameters with a range of 8 to 25 mm. If these criteria were not achieved, aneurysms were deemed as being treated outside of IFU. In comparison, patients within the GREAT registry were deemed to have been treated outside of IFU if the proximal neck length was <15 mm or the infrarenal neck angle was >60^o^. Outcomes between both groups were assessed according to all-cause mortality, aneurysm-related mortality (ARM), all reinterventions, any endoleak, type Ia endoleak, conversions to open repair, and aneurysm rupture.

Statistical analysis

A retrospective analysis of prospectively recorded data from ENGAGE and GREAT was performed. Categorical variables are presented as frequencies with percentages. Continuous variables are presented as mean +/- standard deviation or as median and interquartile range (IQR). The x2 or Fisher exact tests were used for categorical variables, depending on sample size. P-values < 0.05 are considered significant. All statistics were performed using SAS software (SAS Institute, Cary, NC).

## Results

Baseline demographics and AAA characteristics

Across the two studies, a total of 636 patients underwent EVAR for AAA outside of IFU. ENGAGE had 225 cases (responsible for 17.8% of cases included in the previously published analysis) while GREAT had 411 cases (12.4% of the total cohort).

Baseline demographics and anatomic aneurysm characteristics for both cohorts are outlined in Table 1. The mean age was 73 (SD 7.7) years for those treated outside of IFU within ENGAGE while it was 74.9 (SD 7.8) years for those treated outside of IFU in the GREAT registry. Maximum aortic aneurysm diameter was greatest in those treated outside of IFU within the GREAT registry (61.2 mm (SD 14.1) as compared to ENGAGE (60.7 mm (SD 14.5)). Proximal aortic neck length was found to be shorter in those treated with the Excluder stent-graft as compared to the Endurant device (2.2 cm (SD 1.6) vs. 2.5 cm (SD 1.5)). Additionally, infrarenal neck angulation was greatest in the GREAT registry when measured against ENGAGE (60.8^o^ (SD 29.7) vs. 46.5^o^ (SD 32.3)).

**Table 1:** Baseline demographics and anatomic aneurysm characteristics for both cohorts

	Endurant			Excluder		
Baseline characteristics	Out of IFU (n = 225)	Within IFU (n = 1038)	p-value	Out of IFU (n = 411)	Within IFU (n = 2913)	p-value
Age (years)	73 (7.7)	73 (8.2)	.660	74.9 (7.8)	73.2 (8.4)	< .001
Male	182 (80.9)	948 (91.3)	< .001	307 (74.7)	2549 (87.5)	< .001
Anatomical characteristics						
Maximum diameter of the aneurysm (mm)	60.7 (14.5)	60.2 (11)	.675	61.2 (14.1)	56.4 (12.2)	< .001
Proximal aortic neck length (cm)	2.5 (1.5)	2.7 (1.2)	.025	2.2 (1.6)	3.0 (1.6)	< .001
Infrarenal neck angle (degrees)	46.5 (32.3)	26.9 (19.9)	< .001	60.8 (29.7)	25.8 (15.4)	< .001

Outcomes

Mortality, Rupture, and Conversion

Table 2 lists the outcomes. The freedom from all-cause mortality at five years was slightly greater in those treated with the Endurant device (ENGAGE 65.6% vs. GREAT 63.8%). Freedom from aneurysm-related mortality across the same period was 97.2% in the ENGAGE registry while it was 96.2% within the GREAT cohort. The five-year freedom from sac-related rupture was 98.8% in ENGAGE while the requirement for conversion to open repair was 0.4%. Both of these outcomes were not commented upon within the GREAT analysis.

**Table 2:** Postoperative outcomes at five years ENGAGE: Endurant Stent Graft Natural Selection Global Post-Market Registry; GREAT: Global Registry for Endovascular Aortic Treatment; IFU: instructions for use (n/a: not available as assessed analysis did not provide noted endpoint)

	ENGAGE			GREAT		
	Out of IFU, %	Within IFU, %	P-value	Out of IFU, %	Within IFU, %	P-value
	N = 225	N = 1038		N = 411	N = 2913	
Freedom from all-cause mortality (%)	65.6	67.7	.6	63.8	72.5	.002
Freedom from aneurysm-related mortality (%)	97.2	97.9	.39	96.2	98.9	.002
Any endoleak	35.9	30.5	.30	n/a	n/a	n/a
Type Ia	10.6	3.3	< .001	7.0	1.2	< .001
Conversion to open	0.4	2.4	.16	n/a	n/a	n/a
5-year freedom from sac rupture	98.8	98.5	.87	n/a	n/a	n/a
Secondary reintervention	16.0	16.0	n/a	13.3	9.7	.02

Endoleaks and Secondary Procedures

The five-year rate of any endoleak development was assessed for the ENGAGE registry although it was not addressed in the GREAT analysis. This occurred in 35.9% of those treated with the Endurant device. The rate of Ia endoleak development in these two cohorts of patients (treated outside of IFU) was slightly higher with the Endurant device (ENGAGE 10.6% vs. GREAT 7.0%) although this may have been impacted by the fact that only endoleaks that underwent reintervention were recorded within the GREAT registry. Despite the high rate of any endoleak development in the ENGAGE registry, reintervention was relatively similar between the two cohorts (ENGAGE 16% vs. GREAT 13.3%).

## Discussion

This study demonstrates that a significant proportion of patients are undergoing endovascular management of AAA despite failing to satisfy the manufacturer's designated IFU. A multitude of factors are responsible for this, including a lack of regulatory oversight with an increasingly aggressive interventional approach from clinicians. This trend could be attributed to previous data highlighting relatively similar long-term outcomes between those treated endovascularly as compared to OAR although the significantly higher reintervention rate after EVAR must be considered [11-12].

The successful endovascular treatment of an abdominal aortic aneurysm requires the achievement of the exclusion of blood flow to the aneurysm sac with subsequent depressurization of the aneurysmal wall [13]. Previously, aortic neck anatomy has been highlighted as the greatest single predictor of successful intervention [14]. This included factors such as a short aortic neck and a sizeable degree of aortic neck angulation [14]. These features have been well-recognized as risk factors for long-term complications following EVAR, with higher rates of type Ia endoleak development as well as reintervention [15]. They have formed the basis for the IFU for the commercially available grafts. Despite these well-recognized anatomical features, 15.2% (636/4587) of patients across both the ENGAGE and GREAT cohorts underwent treatment outside of IFU.

The newer generation endografts have likely encouraged this trend toward intervention in hostile aortic neck anatomy. The constant development in technology, with the introduction of lower-profile stent-grafts, as well as more liberal IFU, has likely increased the proportion of AAA being managed endovascularly. Despite these noted advancements, stent grafts continue to be utilized outside of IFU as highlighted by this study. This is likely influenced by an increasingly aging and co-morbid population who are likely unsuitable for major open aortic repair. In the absence of alternative options, it seems clinicians are turning to EVAR despite the noted long-term pitfalls of recurrent reinterventions and their associated morbidity.

The present study demonstrates that freedom from all-cause mortality at five years was slightly higher in patients treated with the Endurant device (Medtronic) as compared to the Excluder device (Gore) (65.6% vs. 63.8%). This reflects previously published data that promoted the utilization of active suprarenal fixation, with devices such as the Endurant, in cases with hostile aortic neck anatomy [16]. However, this is not an all-encompassing rule with certain features, such as wide aortic neck diameter, shown to negatively impact outcomes [16]. Freedom from aneurysm-related mortality following the utilization of the Endurant stent graft was 97.2% in comparison to 96.2% with the Excluder endoprostheses. Despite treatment outside of IFU, the treatment modality in question appears to be effective in the primary purpose of preventing aneurysm rupture and death. This is further reinforced by the high five-year freedom from sac rupture outlined in the ENGAGE cohort (98.8%).

Despite the successful deployment of these endoprostheses in preventing aneurysm rupture, the rate of reintervention in this analysis was relatively high (ENGAGE 16% vs. GREAT 13.3%). This has previously been highlighted in the literature with reintervention-free survival estimated in one study to be 86% at three years in those treated outside of IFU, as compared to 96% for those treated within [17]. A further endpoint that is likely representative of the long-term complications following EVAR outside of IFU is the rate of Ia endoleak development in this cohort (ENGAGE 10.6% vs. GREAT 7.0%). This is similar to previously published data that estimated type Ia endoleak as occurring in approximately 3.8%-15% of cases treated outside of the IFU [18]. In response to the high rates of endoleaks and reintervention, further tools have been promoted, including the utilization of endoanchors to assist in proximal stent stabilization. These have been shown to successfully improve the sealing of abdominal endografts in cases of intraoperative type Ia endoleaks in hostile neck anatomies [19]. However, further investigation is required to determine long-term durability in cases where they have been utilized.

Limitations

There are some limitations to this analysis. First, it is a retrospective analysis of an amalgamation of previously published data. However, the data in question (ENGAGE, GREAT) were collected prospectively on an international scale, which provides this analysis with the power of a moderate multicentre study representative of a real-world experience. Second, both ENGAGE and GREAT had a high number of patients lost to follow-up. Once again, this is likely representative of everyday experience with patient factors changing over time. Third, the variability in recorded endpoints between both registries somewhat limits the applicability of these findings. This is highlighted by certain datasets such as endoleak only being recorded in the GREAT registry if it resulted in reintervention. This likely results in an element of bias toward the GREAT analysis.

## Conclusions

Treatment outside of IFU has become increasingly more common with consistent advancements in endograft technology. The increasingly aging and co-morbid population has also led to the promotion of this trend. However, EVAR is fragile to late aortic changes, especially in hostile aortic neck anatomy, with a propensity toward endoleak development and reintervention. This analysis reinforces the importance of the consideration of either fenestrated or open repair in those aneurysms that fail to satisfy IFU while endovascular repair in such a setting should be reserved as a last-resort strategy.

## References

[1] Parodi JC, Palmaz JC, Barone HD (1991). Transfemoral intraluminal graft implantation for abdominal aortic aneurysms. Ann Vasc Surg.

[2] Greenhalgh RM, Brown LC, Kwong GP, Powell JT, Thompson SG (2004). Comparison of endovascular aneurysm repair with open repair in patients with abdominal aortic aneurysm (EVAR trial 1), 30-day operative mortality results: randomised controlled trial. Lancet.

[3] Prinssen M, Verhoeven EL, Buth J (2004). A randomized trial comparing conventional and endovascular repair of abdominal aortic aneurysms. N Engl J Med.

[4] Bertrand M, Godet G, Koskas F, Cluzel P, Fléron MH, Kieffer E, Coriat P (2001). Endovascular treatment of abdominal aortic aneurysms: is there a benefit regarding postoperative outcome?. Eur J Anaesthesiol.

[5] Patel R, Sweeting MJ, Powell JT, Greenhalgh RM (2016). Endovascular versus open repair of abdominal aortic aneurysm in 15-years’ follow-up of the UK endovascular aneurysm repair trial 1 (EVAR trial): a randomised controlled trial. Lancet.

[6] Paravastu SC, Jayarajasingam R, Cottam R, Palfreyman SJ, Michaels JA, Thomas SM (2014). Endovascular repair of abdominal aortic aneurysm. Cochrane Database Syst Rev.

[7] De Bruin JL, Baas AF, Buth J (201020). Long-term outcome of open or endovascular repair of abdominal aortic aneurysm. N Engl J Med.

[8] Oliveira-Pinto J, Oliveira NFG, Bastos-Gonçalves FM (2020). Long-term results after standard endovascular aneurysm repair with the Endurant and Excluder stent grafts. J Vasc Surg.

[9] Mwipatayi BP, Faraj J, Oshin O (2021). Endurant stent graft demonstrates promising outcomes in challenging abdominal aortic aneurysm anatomy. J Vasc Surg.

[10] Barry IP, Barns M, Verhoeven E (2021). Excluder stent graft-related outcomes in patients with aortic neck anatomy outside of instructions for use (IFU) within the Global Registry for Endovascular Aortic Treatment (GREAT): mid-term follow-up results [In Print]. Ann Vasc Surg.

[11] De Bruin JL, Baas AF, Buth J (2010). Long-term outcome of open or endovascular repair of abdominal aortic aneurysm. N Engl J Med.

[12] Greenhalgh RM, Brown LC, Powell JT, Thompson SG, Epstein D, Sculpher MJ (2010). Endovascular versus open repair of abdominal aortic aneurysm. N Engl J Med.

[13] Schanzer A, Greenberg RK, Hevelone N, Robinson WP, Eslami MH, Goldberg RJ, Messina L (2011). Predictors of abdominal aortic aneurysm sac enlargement after endovascular repair. Circulation.

[14] Dillavou ED, Muluk SC, Rhee RY, Tzeng E, Woody JD, Gupta N, Makaroun MS (2003). Does hostile neck anatomy preclude successful endovascular aortic aneurysm repair?. J Vasc Surg.

[15] Antoniou GA, Georgiadis GS, Antoniou SA, Kuhan G, Murray D (2013). A meta-analysis of outcomes of endovascular abdominal aortic aneurysm repair in patients with hostile and friendly neck anatomy. J Vasc Surg.

[16] Tadros RO, Sher A, Kang M (2018). Outcomes of using endovascular aneurysm repair with active fixation in complex aneurysm morphology. J Vasc Surg.

[17] Hwang D, Park S, Kim HK, Lee JM, Huh S (2017). Reintervention rate after open surgery and endovascular repair for nonruptured abdominal aortic aneurysms. Ann Vasc Surg.

[18] Oliveira-Pinto J, Oliveira N, Bastos-Gonçalves F (2017). Long-term results of outside "instructions for use" EVAR. J Cardiovasc Surg (Torino).

[19] Reyes Valdivia A, Beropoulis E, Pitoulias G (2019). Multicenter Registry about the use of endoanchors in the endovascular repair of abdominal aortic aneurysms with hostile neck showed successful but delayed endograft sealing within intraoperative type Ia endoleak cases. Ann Vasc Surg.

